# Whole-Exome Analysis for Polish Caucasian Patients with Retinal Dystrophies and the Creation of a Reference Genomic Database for the Polish Population

**DOI:** 10.3390/genes15081011

**Published:** 2024-08-01

**Authors:** Ewa Matczyńska, Robert Szymańczak, Katarzyna Stradomska, Przemysław Łyszkiewicz, Maria Jędrzejowska, Karolina Kamińska, Marta Beć-Gajowniczek, Ewa Suchecka, Marek Zagulski, Marta Wiącek, Edward Wylęgała, Anna Machalińska, Małgorzata Mossakowska, Monika Puzianowska-Kuźnicka, Sławomir Teper, Anna Boguszewska-Chachulska

**Affiliations:** 1 Genomed S.A., 02-971 Warsaw, Poland; 2 Chair and Clinical Department of Ophthalmology, Faculty of Medical Sciences in Zabrze, Medical University of Silesia, 40-055 Katowice, Poland; 3 First Department of Ophthalmology, Pomeranian Medical University, 70-204 Szczecin, Poland; 4 Study on Ageing and Longevity, International Institute of Molecular and Cell Biology, 02-109 Warsaw, Poland; 5 Department of Human Epigenetics, Mossakowski Medical Research Institute, Polish Academy of Sciences, 02-106 Warsaw, Poland; 6 Department of Geriatrics and Gerontology, Medical Centre of Postgraduate Education, 01-813 Warsaw, Poland; 7 Department of Scientific Research, Branch in Bielsko-Biala, Medical University of Silesia, 43-300 Bielsko-Biała, Poland

**Keywords:** inherited retinal dystrophy, retinitis pigmentosa, population database, WES, WGS

## Abstract

We present the results of the first study of a large cohort of patients with inherited retinal dystrophies (IRD) performed for the Polish population using whole-exome sequencing (WES) in the years 2016–2019. Moreover, to facilitate such diagnostic analyses and enable future application of gene therapy and genome editing for IRD patients, a Polish genomic reference database (POLGENOM) was created based on whole-genome sequences of healthy Polish Caucasian nonagenarians and centenarians. The newly constructed database served as a control, providing a comparison for variant frequencies in the Polish population. The diagnostic yield for the selected group of IRD patients reached 64.9%. The study uncovered the most common pathogenic variants in *ABCA4* and *USH2A* in the European population, along with several novel causative variants. A significant frequency of the *ABCA4* complex haplotype p.(Leu541Pro; Ala1038Val) was observed, as well as that of the p.Gly1961Glu variant. The first *VCAN* causative variant NM_004385.5:c.4004-2A>G in Poland was found and described. Moreover, one of the first patients with the *RPE65* causative variants was identified, and, in consequence, could receive the dedicated gene therapy. The availability of the reference POLGENOM database enabled comprehensive variant characterisation during the NGS data analysis, confirming the utility of a population-specific genomic database for enhancing diagnostic accuracy. Study findings suggest the significance of genetic testing in elder patients with unclear aetiology of eye diseases. The combined approach of NGS and the reference genomic database can improve the diagnosis, management, and future treatment of IRDs.

## 1. Introduction

Inherited retinal dystrophies (IRDs) represent the major group of congenital eye diseases, affecting 1 individual per 1500. They are a very heterogeneous group with at least 50 major types [1]. Similar clinical symptoms can therefore be due to variants in a large number of possible causative genes and, conversely, different genetic variants in the same gene can cause distinct disorders [2].

There is a wide spectrum of IRD phenotypes, from progressive conditions, including those affecting the macula (e.g., Stargardt disease), to stationary conditions such as achromatopsia and congenital stationary night blindness. Retinits pigmentosa (RP) is the most frequent IRD, with a prevalence close to 1:4000 in Europe and worldwide [2,3], encompassing progressive rod-cone dystrophies, characterised by the primary degeneration of rod photoreceptors followed by the cone photoreceptor loss [2]. A gradual loss of the visual field in a concentric pattern may ultimately result in the loss of vision at more advanced stages, with additional, extra-ocular symptoms in 20–30% of cases (syndromic RP) [2].

Most retinal dystrophies are inherited as an autosomal recessive trait, with RP being more frequently dominant [1,2]. There are, however, some genes in which both recessive and dominant inheritance is possible [1]. These dystrophy types can differ in their clinical symptoms. This makes the IRD diagnosis more complex. X-linked inheritance is relatively common, as shown by recent studies [4]. The involvement of the mitochondrial genome in the development of IRD should not be ignored [5]. The mtDNA analysis should therefore be included to search for causative variants in syndromic retinal dystrophies

Increasing application of next-generation sequencing (NGS) methods in genetic diagnostics and decreasing costs of such analyses have allowed for the performance of large studies using whole-exome sequencing (WES), whole-genome sequencing (WGS), and multigene panels. Results of these studies have been recently published and improve our knowledge and our ability to uncover the genetic background of IRD [4,6,7,8]. Over 300 genes are currently known as harbouring pathogenic variants that may cause retinal disease [1].

In response to the high incidence of IRDs and an urgent need for a cure, there are currently numerous gene therapies at various stages of clinical development.

Only one such treatment, Luxturna (voretigene neparvovec, Novartis), was authorised by the FDA (2017) and EMA (2018) to treat RP/Leber’s congenital amaurosis (LCA) patients with pathogenic variants in the *RPE65* gene [9]. In spite of the hopes provoked by the introduction of this treatment, one should not forget that mutations of the *RPE65* gene represent a small percentage of IRDs, while the efficacy of this treatment decreases with the age of the patient and a number of viable retinal cells [10]. Paradoxically, with the development of methods for both gene editing and introducing nucleic acids into the cell using appropriate vectors, it turned out that disease mechanisms are so complex that a simple insertion of the correct gene is an insufficient solution. Hence, an attempt to bypass many obstacles using gene-agnostic methods seems promising and may give hope to patients also at an advanced stage of the disease. Currently, we are observing a dynamic progress in both groups—specific gene-targeted and gene-independent methods [11]. Both will probably be necessary and, moreover, complementary to each other, depending on the type of mutation, retinal morphology, comorbidities, and financial constraints of healthcare systems.

Several genetic studies were carried out for small cohorts of Polish Caucasian IRD patients with Stargardt disease [12,13] and RP [14,15] clinical symptoms. These studies used mostly targeted approaches such as Sanger sequencing [15], SNP microarrays, or small NGS panels, e.g., in LCA [16,17] and cone-rode dystrophy [18] or broader NGS panels [12,13]. The WES approach has been applied in a few studies only [14,19]. Our group performed a large cohort study for another multifactorial retinal degeneration disorder, age-related macular degeneration (AMD), in the frame of the same National Centre for Research and Development grant [20].

Development of diagnostics based on the NGS method (especially using clinical WES and WGS) brings about a need for a well-curated database for the specific population to solve all the ambiguities connected with global databases. The combination of clinical (phenotypic) data with the genotypes is one of crucial requirements for such databases with the intended diagnostic use. Consideration of the best cohort for such purpose leads to the selection of healthy long-lived individuals as the source of genomic reference data. Analysis of the whole-genome data for a precisely selected group of people should also allow an indication of the genetic variants favouring the long-lived phenotype.

Having these two purposes in mind, we focused on the study of whole-genome sequence variation in the Polish population in combination with collected clinical data to create a Polish genomic reference database of extremely long-lived, healthy individuals who, compared to those not genetically predisposed to successful aging and longevity, probably have a lower number of pathogenic variants in genes affecting the risk of various diseases. Therefore, we assumed that such a database would serve as an excellent control for diagnostics of people affected with genetically determined diseases.

The goal of the current project was to identify the most frequent pathogenic variants which may serve as treatment targets in IRD patients. The specific approach adopted, based on WES, should allow to identify pathogenic variants not only in already known genes causing retinal diseases but also in new genes. Moreover, identification of common pathogenic variants would possibly allow for development of new therapies for patients with progressive, currently untreatable retinal dystrophies.

## 2. Materials and Methods

### 2.1. IRD Group and Genetic Testing

This study involved 77 patients with clinical symptoms of retinal dystrophies, recruited by several centres: the Medical University of Silesia in Katowice, Genomed S.A. in Warsaw, and the Pomeranian Medical University in Szczecin (Table S1), and who were included into the cohort between the years 2016 and 2018. The mean age at the time of referral was 40.9 (SD = 16.7).

Patients were subjected to detailed ophthalmological examinations, which involved visual acuity and colour vision testing, autorefractometry, tonometry, perimetry, and dilated fundus examinations, including fundus autofluorescence, optical coherence tomography (OCT), fluorescein angiography, and ERG/EOG, depending on the clinical symptoms. The referring ophthalmologist selected the type of examination for each patient. Results of ophthalmological examinations for a subgroup of retinitis pigmentosa patients were described by Wiacek et al. [19].

Genetic testing was carried out in the laboratory of Genomed S.A., Warsaw, Poland. Genomic DNA was isolated from the peripheral blood using a total DNA isolation kit (Blood Mini kit, A&A Biotechnology, Gdańsk, Poland) according to the manufacturer’s protocol or as described in [20]. Genomic DNA was used as input for constructing an exome-enriched library. Two types of the WES enrichment were used during the study: SureSelectXT Clinical Research Exome or Human Exome V5 (Agilent Technologies, Santa Clara, CA, US) and ClinicalExome (Roche, Basel, Switzerland).

The WES libraries were prepared according to the Agilent or Roche protocols using 100 ng of genomic DNA for each sample.

All the libraries were sequenced using the NextSeq500 or HiSeq4000 (Illumina Inc., San Diego, CA, USA) in the PE150 mode, aiming at the mean target coverage above 100×.

Initial processing of BCL files and demultiplexing was done using the Illumina bcl2fastq. Trimmomatic [21] was applied to trim raw FASTQ files from adapter sequences and low-quality bases. Read mapping to the GRCh37 (hg19) reference genome was performed using the Burrows–Wheeler Alignment tool [22]. Duplicate read pairs were removed with MarkDuplicates (Picard tools package [23]). Alignment files were further processed in accordance with the Genome Analysis Toolkit v3 best practice pipeline [24], and Haplotype Caller was used for variant identification. The quality metrics for the alignment and variants were examined, and all variants within the targeted regions, above the default variant quality threshold, were annotated with Annovar [25]. Further variant filtration and interpretation involved the Gemini framework [26], enabling an analysis of multiple samples in the search for rare pathogenic variants in the whole exome, as well as an in-house variant analysis software (BroVar v2), using the ACMG variant classification guidelines [27,28].

In the first step, targeted exome sequencing data analysis was carried out to identify pathogenic variants in 267 genes associated with retinal dystrophies (based on the RetNet database [1]), and subsequently a search for loss-of-function and likely pathogenic variants of the entire exome sequence was performed for samples with negative results. The result set has been limited to rare variants based on the allele frequencies from 1000 Genomes [29], as well as newly constructed POLGENOM [30] databases. Each rare variant (MAF < 0.01 for 1000 Genomes), with the exception of the known pathogenic variants, was assessed for pathogenicity using multiple in silico predictors, including Alamut ver. 2.9.0 (SOPHiA GENETICS SA, Rolle, Switzerland) with all the incorporated predictors, such as SIFT, Mutation Taster, and Polyphen2. Its presence in variant databases such as ClinVar [31] and HGMD Professional [32] and in the in-house database was checked. Finally, the variant frequency was compared to the expected frequency for the considered disease. Segregation was assessed when possible. Pathogenic and likely pathogenic variants were confirmed using Sanger sequencing if diagnostic results were issued or the quality of NGS data was below expected. Mutation Surveyor V 5.0.1 (Softgenetics, State College, PA, USA) was employed for the Sanger data analysis. Copy number variants were searched for using XHMM [33], and an attempt of copy number variation (CNV) analysis was performed with GermlineCNVCaller from GATKv4 package [34].

A patient case was considered likely solved if one of the confirmatory variants for an autosomal recessive disease was classified as an uncertain significance variant and genotyping results were consistent with clinical data. If such variants were the only ones identified and there were additional data supporting its pathogenicity, a case was also considered likely solved. A partial re-analysis of data was recently performed using updated information on the pathogenicity of presumably causative variants—mainly ClinVar and HGMD Professional—and including re-mapping selected exome datasets to the GRCh38 (hg38) reference genome.

This study was approved by the Ethics Committee of Medical University of Silesia (resolution no. KNW/0022/KB1/105/13, KB 31/2012, and KB/36/A/2013) and adhered to the tenets of The Declaration of Helsinki. Informed written consent was obtained from all the participants.

### 2.2. POLGENOM Group and Genetic Analysis

Recruitment and selection of nonagenarians and centenarians to the Polish Reference Genome for Genomic Diagnostics and Personalised Medicine research project (POLGENOM, alternative acronym PlGen) was described in Skubiszewska et al. [35]. In addition, 23 participants were selected from the POLSTU Project [36] and 58 from the POLSENIOR project [37]. The criteria for successful aging were as follows: age ≥ 90 years; independence or slight dependence in the activities of daily living (the ADL score ≥ 4); no moderate or severe dementia (the MMSE score ≥ 20); no diagnosis of either diabetes, stroke, myocardial infarction, or cancer (basal cell carcinoma and squamous cell carcinoma were allowed); and diagnosis after the age of 80 years. Only 2 cases of myocardial infarction, 2 cases of stroke, 2 cases of diabetes, and 3 cases of cancers (urothelial carcinoma, breast cancer, uterine cancer) were diagnosed at old age. The POLGENOM study participants were not specifically tested for the presence of ophthalmological conditions. The questionnaire checked for the presence or absence of cataracts. Nevertheless, their visual acuity had to be good enough to perform the MMSE test, as well as to warrant independence in everyday living.

In total, 126 genomes (74 women and 52 men) of successfully aging individuals aged ≥ 90 years were sequenced and analysed. The general characteristics of the study group are shown in Table S2.

Genomic DNA was isolated using a salting-out procedure [38]. Whole-genome sequences were obtained using HiSeq 2000 and HiSeq X Ten (Illumina Inc., San Diego, CA, USA) in the PE150 mode, with a mean 30× coverage. An in-house-developed bioinformatic pipeline was used that started by the initial processing of BCL files, along with demultiplexing with the Illumina bcl2fastq, followed by raw FASTQ file trimming from adapter sequences and low-quality bases using Trimmomatic [21] and ending by read mapping to GRCh38 (hg38) with a Burrows–Wheeler Aligner [22]. Alignment files were further processed accordingly to the Genome Analysis Toolkit v3 best practice pipeline [24], with the use of HaplotypeCaller for variant identification, while VEP Ensembl release 83 [39] was applied to annotate sequence variants. Additionally, LOFTEE (version 0.2; Loss-of-function Transcript Effect Estimator), a plugin to VEP, was applied to identify high confidence loss-of-function variants. Variants that had not passed the following filters: coverage sufficient for variant calling in more than 33 samples, location out of low complexity regions, successful GATK variant recalibration, were excluded from further analysis. Selected variants were confirmed using the classical (Sanger sequencing) method.

Mitochondrial genome sequences were also analysed using CLC Genomics Workbench 7 (Qiagen, Qiagen, Hilden, Germany), Mitomaster, MITOMAP, and mtDB Genebank databases [40,41,42]. Identified and categorised variants were added to the POLGENOM database.

### 2.3. POLGENOM Reference Database

The genomic database (https://polgenom.pl/) is supported by one dedicated server, containing a database and application. The Ubuntu LTS version of the Linux operating system was installed on specially selected computer hardware. The database is served by the PostgreSQL engine. The application was written in JavaScript, using the Express/Node.js framework.

## 3. Results

### 3.1. IRD Group

Seventy-seven patients diagnosed with retinal dystrophies underwent genetic testing using the WES approach. The group included 34 (44.2%) women and 43 (55.8%) men, unrelated except for two families, with parent–child duos (Table 1). Patients were divided into two phenotypic groups: the main group included 47 patients with clinical symptoms of retinitis pigmentosa (RP) (61.0%), and the other one included those with unspecific IRD symptoms (30, 39.0%).

### 3.2. POLGENOM Group and Database

The POLGENOM group served as the Polish Caucasian-specific control group for this study. The inclusion criteria into the successfully aging group were fulfilled by 126 individuals (Table S2), for whom the whole-genome sequencing and bioinformatic analysis were carried out, and whose genomic variants were used to create the database records.

#### 3.2.1. Statistics

Almost 16.7 million variants in the nuclear genome of the study participants were found (22 million before filtering), including 15.3 million SNVs and 1.4 million small insertions and deletions (InDels). A total of 13.6 million (81%) variants have been already present in the dbSNP database (build 147 and 148), 12.3 million (73.4%) in the 1000 Genomes Project (1000G, Phase 3). Almost 2.76 million novel SNVs and 0.34 million novel indels were detected (database record on 18 February 2019).

The POLGENOM project results were also compared with the current version of the GnomAD database (v4.1.0) (https://gnomad.broadinstitute.org), revealing 1,197,199 novel variants found in the Polish population, of which 1,149,439 (96%) are SNVs, 13,631 small insertions, and 34,129 small deletions.

#### 3.2.2. Mitochondrial Data

For each long-lived individual, a list of mtDNA variants with their respective heteroplasmy level and haplogroup assignment was obtained. The identified variants were classified based on their frequency in the GenBank and mtDB databases, localisation, functional effect, and clinical data (MitoMaster, MITOMAP). In total, there were 215 variants with <0.5% frequency in mtDB or newly identified that were included into the database.

### 3.3. Molecular Diagnosis of IRD

In total, a definitive molecular diagnosis was reached in 45 (58.44%) cases, while 5 cases could be considered as likely solved, which resulted in total of 50 solved and likely solved cases (64,94%). For seven patients, results were inconclusive. For 20 patients, causative variants could not be found, and only carriership for pathogenic or likely pathogenic variants was detected. Positive cases among RP patients accounted for 63.8%, and for the unspecific IRD group, 66.6%.

**Figure 1:**
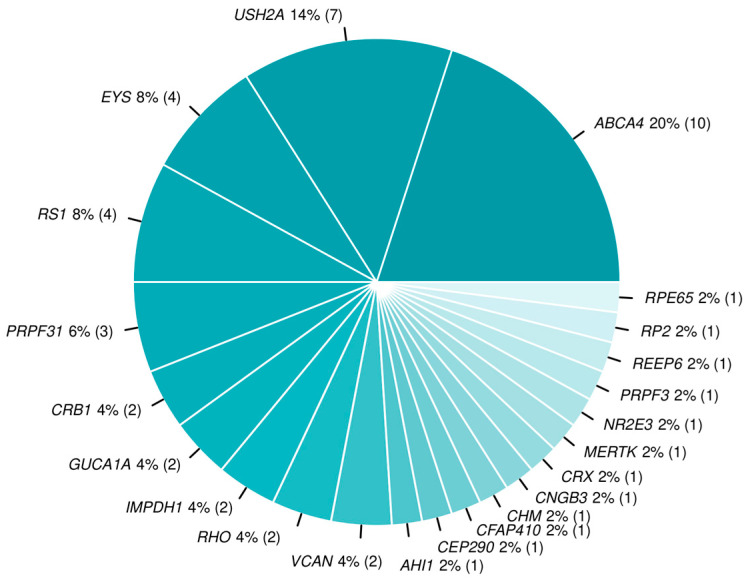
Distribution of genes with confirmatory variants among 50 distinct solved patients (total number of patients with confirmatory variants in the denoted gene in brackets).

**Figure 2:**
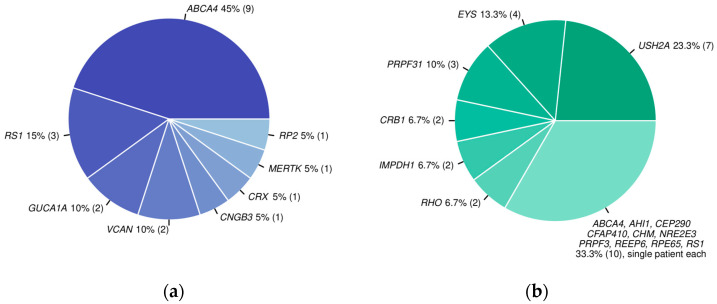
Distribution of genes with confirmatory variants among distinct solved patients in the two groups: (**a**) unspecific IRD group; (**b**) RP group. Total number of patients with confirmatory variants in the denoted gene in brackets.

Confirmatory variants were found in 22 distinct genes (Table 2). The largest group of patients had causative variants in the *ABCA4* gene (10, 20%), followed by *USH2A* (7, 14%), *EYS* (4, 8%), and *RS1* (4, 8%) (Figure 1). A total of 11 (22%) patients presented causative variants in genes that were identified only once in our group. Looking at the distributions of confirmatory genes for both groups, namely, patients with indication of RP and the unspecific IRD group (Figure 2), a predominance of patients with pathogenic variants identified in *ABCA4* was clearly visible in the unspecific IRD group, whereas *USH2A*, *EYS*, and *PRPF31* genes contributed the most to the genetic diagnosis of patients in the RP group.

Pathogenic or likely pathogenic variants were identified predominantly in genes already known to cause retinal degenerations (with a significant number of *ABCA4* variants), although variants in genes newly identified as involved in retinal disorders were also uncovered (such as *CFAP410* and *GUCA1A*). A new clinical diagnosis could be suggested in three cases with the initial indication of RP (*CHM*, *AHI1*, *RS1*—single case each), as well as in five cases from the Unspecific IRD group, where a new diagnosis was found due to causal variants in the *VCAN* and *RS1* genes (two cases and three cases, respectively) (Table 1).

The *ABCA4* (NM_000350.3, NP_000341.2) complex haplotype p.(Leu541Pro; Ala1038Val) was observed in three patients, while the hypomorphic variant (c.5603A>T p.Asn1868Ile) was observed in two patients. All these patients were referred for genetic diagnostics at an older age (45 years and more, Table S1), confirming the correlation of these variants with a milder form of late-onset macular dystrophy.

Missense variants corresponded to 49% of the identified confirmatory variants, frameshift variants to 19%, stopgain variants to 18%, and splicing ones to 11%. One of the few splicing variants identified in the study group was a canonical splice site variant NM_004385.5:c.4004-2A>G in *VCAN*. This transition presumably modifies the 3′ acceptor splice site of intron 7 and activates a cryptic exon 8 splice site, 39 bp downstream from the authentic one [43,44]. In one patient, two missense variants (NM_000329.3:c.131G>A p.Arg44Gln and NM_000329.3:c.709C>T p.Pro237Ser) in the *RPE65* gene were uncovered, the first one being a well-characterised pathogenic variant while the second one a likely pathogenic variant with only one record in ClinVar but with strong predictions of pathogenicity, obtained using a set of prediction programmes. Both variants change highly conserved amino acids, affecting the *RPE65* protein function. In one patient, two missense variants in the *AHI1* gene (NM_017651.5, NP_060121.3) were indicated as the most probable causative variants (c.1828C>T p.Arg610Ter and c.1829G>C p.Arg610Pro), indicating a possible genetic diagnosis of Joubert Syndrome type 3.

Seven novel variants identified in our cohort are still not present in any databases (Table 2). They include missense variants in *PRPF31* (NM_015629.4:c.831T>A p.Ser277Arg) and in *USH2A* (NM_206933.4:c.10732A>C p.Ser3578Arg); frameshift variants in *ABCA4* (c.2548dup p.Tyr850LeufsTer9), in *EYS* (NM_001142800.2:c.4462_4469dup p.Met1491AlafsTer12), and *PRPF31* (NM_015629.4:c.428_430delins22ACAAGTGCAAGGCTGTTCTTGC p.Gly143AspfsTer17); and a splicing variant in *CHM* (NM_000390.4:c.49+2T>A). Of those, the *ABCA4* and *EYS* variants have been subsequently uncovered in a single patient each, while the *USH2A* variant was found twice in our further IRD panel studies (Matczyska et al. [45]).

The POLGENOM database served as a reference database in the bioinformatic analysis of data. Data on the POLGENOM frequency of IRD causative variants, identified in this study, are shown in Table 2. Interestingly, only the predominant *ABCA4* variant in our study group (found in five patients, always in compound heterozygosity with another variant), c.5882G>A p.Gly1961Glu, and the hypomorphic variant, c.5603A>T, p.Asn1868Ile (found in two patients, always in compound heterozygosity with a severe pathogenic variant), were present.

Among the most interesting unsolved cases was a family duo in which two VUS variants of a possible AD effect were uncovered (in *AIPL1* and *RP1*) with the second variant that could possibly influence the clinical phenotype (Table S1). This seems to be a case that cannot be solved without a deeper analysis of all the family members, including a search for CNVs.

### 3.4. Selected Genetic Diagnosis Cases

Using the WES approach for the recruited group of the IRD patients, a few rare causative genes and treatable disease cases were uncovered. VUS variants corresponding to clinical symptoms were reported as likely causative due to their absence from population databases and indications of prediction programmes suggesting their strong pathogenicity. As the diagnostic process is always based on collaboration between a molecular geneticist and a referring physician, the final diagnostic decision is made by an ophthalmogenetic specialist.

#### 3.4.1. Retinitis Pigmentosa Case (Compound Heterozygote in *RPE65*)

The young (14-year-old) male patient, for whom two missense variants in the *RPE65* gene were uncovered, reported the following symptoms: narrow field of vision, decreased visual acuity, and poor night vision. The patient also had myopic astigmatism. Best corrected visual acuity: OD 0.1; OS: 0.3 by Snellen. In May 2023, he received treatment with Luxturna (voretigene neparvovec, Spark Therapeutics/Novartis) in the eye with lower visual acuity, which allowed for the stabilisation of retinal morphology, improvement in night vision, and reduced photophobia.

#### 3.4.2. Wagner Syndrome - Family Case (Heterozygote in *VCAN*)

**Figure 3:**
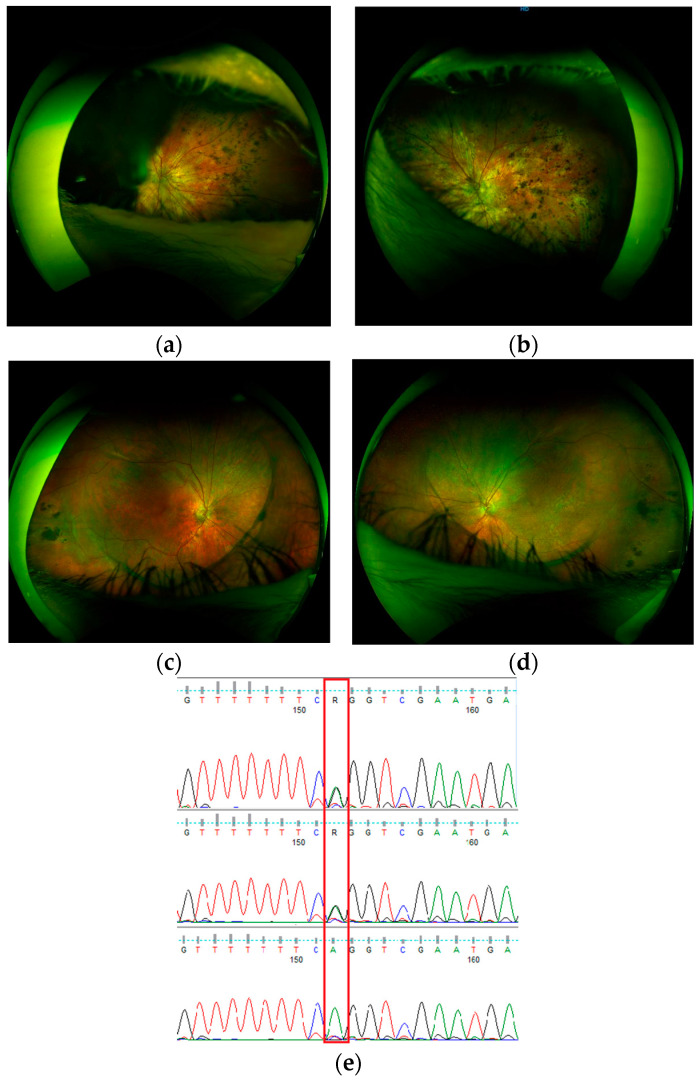
Ultrawidefield eye fundus images (Optos) - VCAN-related Wagner syndrome family: patient S48, father, 52 years old: (**a**) (right eye, RE), (**b**) (left eye, LE); patient S46, son, 20 years old (**c**) (LE), (**d**) (RE); (**e**) Sanger confirmation of the NM_004385.5:c.4004-2A>G variant in *VCAN* for patient S46 (top), patient S48 (middle), and wild-type control sample (bottom). Due to changes involving the anterior segment of the eye in the father, with extensive phimosis of the lens capsule after cataract surgery, fundus imaging is difficult. In this case, widefield images cover a limited area of the retina; in the right eye, only the nasal part of the retina is visible (**a**). Pigmentary changes located mainly on the periphery of the retina resemble retinitis pigmentosa, which was the first, initial diagnosis in both father and son. Visual acuity was low at 0.01 (RE), 0.05 (LE), but stable for the last 10 years. In his son, pigmentary and atrophic changes were less severe. The boundary line of the optically empty part of the vitreous body was clearly marked, from which vitreoretinal tractions extend towards the periphery. Best corrected visual acuity is 1.0 (RE) and 0.9 (LE), but with a narrowed field of vision. Snellen charts were used for visual acuity examination.

Wagner syndrome was detected for the first time in Poland in a family previously diagnosed as having dominant retinitis pigmentosa (Figure 3). Wagner syndrome, also known as dominant hyaloideoretinal dystrophy or VCAN-related vitreoretinopathy, is a hereditary eye disorder that leads to progressive vision loss. The vitreous becomes thin and watery, appearing empty. Retinal tractions and tears can result in the retinal detachment. Wagner syndrome is associated by ophthalmologists primarily with the above-mentioned structural changes of the vitreous body. Therefore, in patients who do not present with early retinal detachment, diagnosis may be difficult. Other signs and symptoms are decreased visual acuity (often associated with lens opacities), progressive night blindness, and a narrowing visual field, as well as thinned retina and choroid, pigment changes in the periphery of the retina, and slightly reduced thickness of retinal vessels, which all may suggest retinitis pigmentosa.

Wagner syndrome is caused by genetic changes in the *VCAN* gene (previously named *CSPG2*), which encodes Versican protein, a chondroitin sulphate proteoglycan-2 found in the vitreous among other tissues. It is an extremely rare disease, with not much more than 250 patients having been described to date (together with another disease due to mutations in the *VCAN* gene-erosive vitreoretinopathy) [44]. The canonical splice site variant NM_004385.5:c.4004-2A>G in *VCAN*, found in this study, was previously reported only in two families [44,45], while, based on the HGMD records, splicing mutations in intron 7 and 8 as well as deletions of the entire exon 8 or of its part are the main causes of Wagner syndrome.

## 4. Discussion

We attempted one of the first and the widest WES-based characterisations of variants causing inherited retinal dystrophies in the Polish Caucasian population.

To facilitate all types of high-throughput genetic testing for the Polish Caucasian population, a Polish Genomic Database was created, including WGS sequences together with biochemical data and health records of 126 health nonagenarians and centenarians.

Such databases have been created for other populations, beginning from the Icelandic one [46], and currently the TopMed [47] and the UK project [48], but rarely for a selected group of healthy, long-lived people, gathering additional, easily accessible data on their individual health status. The first data from a larger Polish whole-genome project were published in 2022 [49]. Its participants were recruited by several medical centres to study the genetic susceptibility to COVID-19 infections, and therefore the cohort is not representative of the entire Polish population. Moreover, these data are currently not available for other projects and diagnostic purposes while the POLGENOM database, a homogenous whole-genome database of healthy extremely age-advanced individuals, remains the only accessible reference genomic database for the Polish Caucasian population. It is available at https://polgenom.pl/ for any type of project requiring assembled genetic and health status records for the Caucasian population.

This combined approach allowed us to provide molecular confirmation of clinical diagnosis in 50 (64.94%) cases and establish possible genetic aetiology in 7 cases out of 77 WES-analysed individuals. This result was an improvement in regard to the initial conclusions, owing to the partial re-analysis of the variant lists, taking into account new data on the pathogenicity of variants, especially those in the *ABCA4* gene. The data re-analysis led us also to the re-classification of three variants from VUS to likely benign and removal of these cases from the list of possibly solved cases. The final diagnostic yield, however, is comparable to the results of other NGS-based studies of IRD in different populations. The diagnostic success rate varied between 51 and 76% [4,6,7,8], depending on the approach adopted (NGS panel/WES/WGS), availability of CNV analysis, and the study group composition. Interestingly, the highest success rate reported for WES was 89% for 19 well-characterised non-syndromic Polish RP patients, owing to a large set of clinical/family data; extensive feedback from an ophthalmogenetic specialist; and an improved CNV analysis based on consistent data from a newer, fine-tuned exome enrichment [14].

The prevalence of confirmatory *ABCA4* variants, noted in the unspecific IRD group, correlated with the higher referral age of patients (Table S3), is of importance for diagnostics and medical care for elder patients with eye disease problems of unclear aetiology, suggesting a need for genetic testing even at a more advanced age. A diagnosis of a late-onset genetic disorder may allow for the avoidance of unnecessary treatment with a risk of serious adverse effects, especially since differential diagnosis often includes inflammatory diseases requiring immunosuppressive or biological agents.

This was the first WES-based study of a large group of patients with inherited eye diseases initiated in Poland. The introduction of a new diagnostic method, based on the whole-exome analysis, encouraged people who had not previously decided to use genetic diagnostics, especially in diseases considered difficult to confirm using classical methods. Interestingly, this may apply to situations where a specific gene (or set of genes) is strongly suspected as the cause of the disease, but a slight change in the phenotype compared to the descriptions of the disease in scientific publications may mislead the initial diagnosis. This way, for the first time in Poland, Wagner syndrome was detected and genetically confirmed in a family previously diagnosed as having dominant retinitis pigmentosa. The identification of the genetic background of the disease allowed for the provision of specific lifestyle advice, a warning against the symptoms of retinal detachment, and a discontinuation of unnecessary medications.

For the patient with the *RPE65* mutations, a gene therapy could be started with positive results two months after its application. Other patients with positive genetic results, including pathogenic variants in most frequently mutated genes (such as *ABCA4*, *EYS, USH2A*), new genetic counselling options and new therapies will be available soon, with genome editing as one of the options.

Among future gene therapies for RP, there is a combination of gene therapy and so-called optogenetics, with two complementary components: a construct, encoding a photoactivatable channel rhodopsin protein, delivered via a modified adenovirus vector, and biomimetic goggles that stimulate the engineered retinal cells [50,51]. A phase I/II trial was successfully ended for intravitreal antisense oligonucleotide sepofarsen [52] to treat LCA type 10, caused by *CEP290* mutations. Another advanced and positive gene therapy clinical trial is that for patients with LCA type 1 caused by mutations in *GUCY2D* [53]. Treatment of *LCA5* gene-dependent LCA (type 5) is similarly advanced [54], while a so-called “molecular photoswitch”, conferring light-sensing capabilities to special types of retinal neuron cells, could potentially become a new treatment in RP and choroideremia [55]. Future treatment of IRD will, however, probably rely on CRISPR-Cas9-mediated gene editing either in human-induced pluripotent stem cells derived from patients and subsequent subretinal transplantation of differentiated retinal cells, or in vivo, by subretinal injection of a modified adenovirus vector carrying a CRISPR-Cas9-guide RNA-expressing construct. Successful results in cell cultures were achieved for several types of retinal dystrophies such as Stargardt disease [56] and LCA [57]. For patients with the most common *CEP290* mutation, a gene editing treatment is already available in frame of a clinical trial [58]. A gene-agnostic treatment—an antioxidant nanotherapeutic—could also be available soon [59]. It has been proposed for diseases affecting the posterior segment of the eye and it consists of polycaprolactone nanoparticles delivering anti-oxidative drugs to the retina with a durable effect.

Possible reasons of unsolved cases or nonconclusive results in our study are the following, based on our current experience: exome enrichments still under development—in consequence, GC-rich regions, regions with tandem repeats, and genes with pseudogenes have not been efficiently sequenced; bioinformatic pipelines were still under development during the study period; inefficient identification of CNV and of deep intronic variants; not enough data on variant pathogenicity, with databases and tools still under development; data missing on new genes involved in retinal degeneration; and the study group was too small to identify new IRD genes [6,7,45].

Taking into account the presented results, we focused our efforts on developing an optimised NGS panel, because it allowed for an overall increase in uniformity and level of coverage at a lower cost (along with an improved coverage in cumbersome regions, such as the *RPGR* ORF15), supporting a successful CNV analysis [45]. This approach enabled us to reach the final molecular diagnosis in one of the patients of this cohort (Table S1, patient S42). A customised exome enrichment with additional probes improving CNV identification and sequencing of deep intron variants could be an option with a similar diagnostic yield. Surely, the WGS approach should allow for up to 10% increase in molecular diagnoses when compared to WES and targeted approaches as a consequence of a more homogeneous coverage. It has not only a much higher potential for identifying structural variants such as translocations and inversions due to significantly more complete analysis of breakpoints but also allows for a more comprehensive sequencing of variants in non-coding regions [6].

## 5. Conclusions

Our results confirm that over 60% IRD patients referred for genetic testing by ophthalmologists and clinical geneticists with experience in IRD diagnosis can expect a positive result, based on WES analysis limited to the RetNet scope. Causative variants for an extremely rare disease (Wagner syndrome) as well as those in the gene being a target of one of the first gene therapies (*RPE65* and Luxturna) were able to be uncovered using this WES approach, still under development a few years ago, supported by the availability of a reference population genomic database.

The results of this study supported, however, the development of a targeted retinal panel that would cover most pathogenic variants occurring in the Polish population, and would allow for a fast and low-cost genetic analysis. Along the diagnostic algorithm proposed by our group (Matczyńska et al. [45]), such a targeted approach can be followed by a wider dystrophy panel, based on WES or WGS for unsolved cases, eventually leading to the final diagnosis and selection of a personalised therapy. The POLGENOM database remains a constant support for such types of analysis.

## Figures and Tables

**Table 1:** IRD group characteristics.

Initial Indication	Total Cases	Females	Males	Mean Age (SD)	Solved and Likely Solved Cases	New Clinical Diagnosis
RP	47	19 (40.4%)	28 (59.6%)	38.26 (SD = 14.55)	30 (63.8%)	3
Unspecific IRD	30	15 (50.0%)	15 (50.0%)	46.04 (SD = 19.56)	20 (66.7%)	5
Total	77	34	43	40.93 (SD = 16.71)	50 (64.9%)	8

**Table 2:** List of causative and possibly causative variants found in the analysed cohort of 77 IRD patients (LP—likely pathogenic).

Gene	Variant Class	HGVSc	HGVSp	No Patients	No Alleles	POLGENOM Frequency	gnomAD_2.1.1.AF Total	dbSNP	Clinvar ID	Consequence	Novel	P/LP/VUS
*ABCA4*	SNV	NM_000350.3:c.214G>A	NP_000341.2:p.Gly72Arg	1	1		0.00001	rs61751412	99120	missense		Pathogenic
*ABCA4*	SNV	NM_000350.3:c.1622T>C	NP_000341.2: p.Leu541Pro	4	5	-	0.00016	rs61751392	99067	missense		Pathogenic
*ABCA4*	SNV	NM_000350.3:c.3113C>T	NP_000341.2:p.Ala1038Val	4	5	-	0.00175	rs61751374	7894	missense		Pathogenic
*ABCA4*	InDel	NM_000350.3:c.2548dup	NP_000341.2:p.Tyr850LeufsTer9	1	1	-	-	-		frameshift	yes	Pathogenic
*ABCA4*	SNV	NM_000350.2:c.2588G>C	NP_000341.2:p.Gly863Ala	2	2	-	0.00430	rs76157638	7879	missense		Pathogenic
*ABCA4*	SNV	NM_000350.3:c.4234C>T	NP_000341.2:p.Gln1412Ter	1	1	-	0.00001	rs61750137	99263	stop_gained		Pathogenic
*ABCA4*	InDel	NM_000350.3:c.4537dup	NP_000341.2:p.Gln1513ProfsTer42	1	1	-	0.00002	rs281865377	99292	frameshift		Pathogenic
*ABCA4*	SNV	NM_000350.3:c.5189G>A	NP_000341.2:p.Trp1730Ter	1	1	-	0.00000	rs886044747	236125	stop_gained		Pathogenic
*ABCA4*	SNV	NM_000350.3:c.5318C>T	NP_000341.2:p.Ala1773Val	1	1	-	0.00001	rs760549861	236129	missense		Pathogenic
*ABCA4*	SNV	NM_000350.3:c.5603A>T	NP_000341.2:p.Asn1868Ile	2	2	0.0635	0.04219	rs1801466	99390	missense		Hypomorphic
*ABCA4*	SNV	NM_000350.3:c.5882G>A	NP_000341.2:p.Gly1961Glu	5	5	0.0119	0.00456	rs1800553	7888	missense		Pathogenic
*AHI1*	SNV	NM_001134831.2:c.1828C>T	NP_001128303.1:p.Arg610Ter	1	1	-	0.00001	rs751734985	984718	stop_gained		Pathogenic
*AHI1*	SNV	NM_001134831.2:c.1829G>C	NP_001128303.1:p.Arg610Pro	1	1	-	-	rs374009466	802272	missense		VUS
*CEP290*	SNV	NM_025114.4:c.4882C>T	NP_079390.3:p.Gln1628Ter	1	1	-	0.00007	rs376493409	217635	stop_gained		Pathogenic
*CEP290*	SNV	NM_025114.4:c.5254C>T	NP_079390.3:p.Arg1752Trp	1	1	-	0.00002	rs748471942	866339	missense		LP
*CFAP410*	InDel	NM_004928.3:	NP_004919.1:p.Ala12SerfsTer60	1	1	-	0.00008	rs748531024	438159	frameshift		Pathogenic
		c.33_34insAGCTGCACAGCGTGCA										
*CFAP410*	SNV	NM_004928.2:c.218G>C	NP_004919.1:p.Arg73Pro	1	1	-	0.00033	rs140451304	428573	missense		LP
*CHM*	SNV	NM_000390.4:c.49+2T>A		1	1	-	-	-		splice_donor	yes	Pathogenic
*CNGB3*	SNV	NM_019098.5:c.1578+1G>A		1	2	-	0.00002	rs372006750	189031	splice_donor		Pathogenic
*CRB1*	SNV	NM_201253.3:c.2042G>A	NP_957705.1:p.Cys681Tyr	1	1	-	0.00000	rs62636266	99874	missense		Pathogenic
*CRB1*	SNV	NM_201253.3:c.2171A>G	NP_957705.1:p.Tyr724Cys	2	2	-	0.00001	rs765676754	872033	missense		Pathogenic
*CRB1*	SNV	NM_201253.3:c.2843G>A	NP_957705.1:p.Cys948Tyr	1	1	-	0.00013	rs62645748	39614	missense		Pathogenic
*CRX*	SNV	NM_000554.6:c.766C>T	NP_000545.1:p.Gln256Ter	1	1	-	-	rs1968173024	861103	missense		LP
*EYS*	SNV	NM_001142800.2:c.2055T>A	NP_001136272.1:p.Cys685Ter	1	1	-	-	rs372354156	194194	stop_gained		Pathogenic
*EYS*	SNV	NM_001142800.2:c.2259+1G>A		1	1	-	0.00004	rs752736741	558511	splice_donor		Pathogenic
*EYS*	InDel	NM_001142800.2:c.4350_4356del	NP_001136272.1:pIle1451ProfsTer3	1	2	-	0.00008	rs761238771	195936	frameshift		Pathogenic
*EYS*	InDel	NM_001142800.2:c.4462_4469dup	NP_001136272.1:p.Met1491AlafsTer12	1	1	-	-	-		frameshift	yes	Pathogenic
*EYS*	SNV	NM_001142800.2:c.4955C>G	NP_001136272.1:p.Ser1652Ter	1	1	-	-	rs909730457	867110	stop_gained		Pathogenic
*EYS*	SNV	NM_001142800.2:c.8816G>A	NP_001136272.1:p.Cys2939Tyr	1	1	-	-	rs1582139965	1039939	missense		VUS
*EYS*	InDel	NM_001142800.2:c.9079_9082del	NP_001136272.1:p.Arg3027SerfsTer5	1	1	-	-	rs1427770112	556940	frameshift		LP
*GUCA1A*	SNV	NM_001384910.1:c.320T>C	NP_000400.2:p.Ile107Thr	2	2	-	-	rs869320710	225233	missense		LP
*GUCY2D*	InDel	NM_000180.4:c.2944+1del		1	1	-	0.00036	rs61750185	98582	splice_donor		Pathogenic
*IMPDH1*	SNV	NM_000883.4:c.730A>C	NP_000874.2:p.Thr244Pro	1	1	-	0.00000	rs1253235754	845744	missense		VUS
*IMPDH1*	SNV	NM_000883.4:c.931G>A	NP_000874.2:p.Asp311Asn	1	1	-	-	rs121912550	14834	missense		Pathogenic
*MERTK*	SNV	NM_006343.2:c.79G>T	NP_006334.2:p.Glu27Ter	1	2	-	-	rs1223798126		stop_gained		Pathogenic
*NR2E3*	InDel	NM_014249.4:c.481del	NP_055064.1:p.Thr161HisfsTer18	1	2	-	0.00007	rs759339012	636047	frameshift		Pathogenic
*PRPF3*	SNV	NM_004698.2:c.1481C>T	NP_004689.1:p.Thr494Met	1	1	-	-	rs121434241	3352	missense		Pathogenic
*PRPF31*	InDel	NM_015629.4: c.428_430delins22AC-	NP_056444.3: p.Gly143AspfsTer17	1	1	-	-	-		frameshift	yes	LP
		AAGTGCAAGGCTGTTCTTGC										
*PRPF31*	InDel	NM_015629.4:c.808dup	NP_056444.3:p.His270ProfsTer9	1	1	-	-	rs2073875313	865752	frameshift		LP
*PRPF31*	SNV	NM_015629.4:c.831T>A	NP_056444.3:p.Ser277Arg	1	1	-	-	-		missense	yes	VUS
*REEP6*	SNV	NM_138393.4:c.349-1G>T		1	1	-	-	rs2085005383	958464	splice_acceptor		LP
*REEP6*	SNV	NM_001329556.3:c.367T>C	NP_001316485.1:p.Cys123Arg	1	1	-	0.00001	rs1341620671		missense		VUS
*RHO*	SNV	NM_000539.3:c.560G>A	NP_000530.1:p.Cys187Tyr	1	1	-	-	rs1578280588	812397	missense		Pathogenic
*RHO*	SNV	NM_000539.3:c.1040C>T	NP_000530.1:p.Pro347Leu	1	1	-	0.00000	rs29001566	13014	missense		Pathogenic
*RP2*	InDel	NM_006915.3:c.14_16del	NP_008846.2:p.Phe5del	1	1	-	-	rs1556313414	437942	inframe deletion		Pathogenic
*RPE65*	SNV	NM_000329.3:c.131G>A	NP_000320.1:p.Arg44Gln	1	1	-	0.00004	rs61751282	98840	missense		Pathogenic
*RPE65*	SNV	NM_000329.3:c.709C>T	NP_000320.1:p.Pro237Ser	1	1	-	-	-	3028862	missense		LP
*RS1*	SNV	NM_000330.4:c.214G>A	NP_000321.1:p.Glu72Lys	1	1	-	0.00001	rs104894928	9888	missense		Pathogenic
*RS1*	SNV	NM_000330.4:c.440G>A	NP_000321.1:p.Trp147Ter	1	1	-	-	-		missense	yes	LP
*RS1*	InDel	NM_000330.4:c.522+1G>T		1	1	-	-	rs281865348	98980	splice_donor		Pathogenic
*RS1*	SNV	NM_000330.4:c.625C>T	NP_000321.1:p.Arg209Cys	1	1	-	-	rs281865361	99006	missense		Pathogenic
*USH2A*	InDel	NM_206933.4:c.775_776del	NP_996816.3:p.Ser259PhefsTer63	1	1	-	-	rs2038566220	866168	frameshift		Pathogenic
*USH2A*	InDel	NM_206933.4:c.1836_1839dup	NP_996816.3:p.Gly614TyrfsTer6	1	1	-	-	rs2102610607	1075936	frameshift		Pathogenic
*USH2A*	SNV	NM_206933.4:c.2276G>T	NP_996816.3:p.Cys759Phe	1	1	-	0.00097	rs80338902	2356	missense		Pathogenic
*USH2A*	SNV	NM_206933.4:c.4210G>T	NP_996816.3:p.Glu1404Ter	1	1	-	-	rs2034849647	813119	stop_gained		Pathogenic
*USH2A*	SNV	NM_206933.4:c.5530C>T	NP_996816.3:p.Gln1844Ter	1	1	-	-	rs761075303	1065688	stop_gained		Pathogenic
*USH2A*	InDel	NM_206933.4:c.5865_5866del	NP_996816.3:p.Ser1956CysfsTer16	1	1	-	-	rs281865361	3028715	frameshift		LP
*USH2A*	SNV	NM_206933.4:c.9424G>T	NP_996816.3:p.Gly3142Ter	1	1	-	0.000024	rs397518048	48626	stop_gained		Pathogenic
*USH2A*	SNV	NM_206933.4:c.10732A>C	NP_996816.3:p.Ser3578Arg	1	1	-	-	-		missense	yes	LP
*USH2A*	SNV	NM_206933.4:c.11864G>A	NP_996816.3:p.Trp3955Ter	2	3	-	0.00012	rs111033364	2357	stop_gained		Pathogenic
*USH2A*	SNV	NM_206933.4:c.12284G>A	NP_996816.2:p.Gly4095Asp	1	1	-	-	rs759898765	378847	missense		Pathogenic
*USH2A*	InDel	NM_206933.4:	NP_996816.3:	2	2	-	-	rs1553252388	438013	inframe_indel		Pathogenic
		c.13335_13347delinsCTTG	p.Glu4445_Ser4449delinsAspLeu									
*VCAN*	SNV	NM_004385.5:c.4004-2A>G		2	2	-	-	rs80356555	17494	splice_acceptor		Pathogenic
**Variants in inconclusive results**												
*AIPL1*	SNV	NM_014336.5: c.737A>C	NP_055151.3: p.Tyr246Ser	2	2	-	0.00021	rs138585919	324614	missense		VUS
*CAPN5*	SNV	NM_004055.5:c.64C>T	NP_004046.2:p.Arg22Cys	1	1		0.00007	rs781837640	1064369	missense		VUS
*HMCN1*	SNV	NM_031935.3: c.16034A>G	NP_114141.2: pGln5345Arg	1	1		0.00071	rs121434382	2205	missense		VUS/Risk factor
*PRPH2*	SNV	NM_000322.5:c.94A>G	NP_000313.2: p.Ile32Val	1	1	-	0.00081	rs61755767	98722	missense		VUS
*RP1*	SNV	NM_006269.2:c.6338C>A	NP_006260.1: p.The2113Asn	2	2	-	0.00022	rs137887415	847529	missense		VUS
*SEMA4A*	SNV	NM_022367.4:c.241C>G	NP_071762.2: p.Arg81Gly	1	1	-	-	rs745715951		missense		VUS

## Data Availability

All necessary data are included in the Supplementary Materials. Please contact the corresponding author to discuss the possibility of obtaining additional data or detailed information.

## Supplementary Materials

The following supporting information can be downloaded at https://www.mdpi.com/article/10.3390/genes15081011/s1. Table S1: List of 77 IRD patients along with the confirmatory/VUS variants. Table S2: General characteristics of the POLGENOM group. Table S3: Genes with confirmatory variants grouped by patients’ age quartile.
