# Supplementary material for: Whole-Exome Analysis for Polish Caucasian Patients with Retinal Dystrophies and the Creation of a Reference Genomic Database for the Polish Population
Source: Genes (Basel). 2024 Aug 1;15(8):1011. doi: 10.3390/genes15081011 (PMC11353931; doi:10.3390/genes15081011)
Supplement: Supplementary file 1 [file genes-15-01011-s001.zip › Table_S2_Characteristics_of_the_study_group_POLGENOM.pdf]

Characteristics of the study group: nonagenarians and centenarians		All	Women	Men
Sex		126	74 (58.7%)	52 (41.3%)
Age*	[yrs]	96.8 [91.0-100.2]	98.1 [91.2-100.8]	94.6 [90.7-99.6]
Height*	[cm]	154 [148-162]	150 [146-154]	163 [160-168]
Weight*	[kg]	60 [52-72]	57 [49-62]	72 [63-81]
BMI*		25.7 [23.2-28.0]	25.5 [22.9-26.8]	26.3 [24.3-29.1]
smoking	Yes	0	0	0
	In the past	30 (23.8%)	5 (6.8%)	25 (48.1%)
	No	96 (76.2%)	69 (93.2%)	27 (51.9%)
Mean No of variants		4 855 580	4 888 158	4 809 219

*Median[Q1-Q3]
